# An Updated Review of Computer-Aided Drug Design and Its Application to COVID-19

**DOI:** 10.1155/2021/8853056

**Published:** 2021-06-24

**Authors:** Arun Bahadur Gurung, Mohammad Ajmal Ali, Joongku Lee, Mohammad Abul Farah, Khalid Mashay Al-Anazi

**Affiliations:** ^1^Department of Basic Sciences and Social Sciences, North-Eastern Hill University, Shillong, 793022 Meghalaya, India; ^2^Department of Botany and Microbiology, College of Science, King Saud University, Riyadh 11451, Saudi Arabia; ^3^Department of Environment and Forest Resources, Chungnam National University, 99 Daehak-ro, Yuseong-gu, Daejeon 34134, Republic of Korea; ^4^Department of Zoology, College of Science, King Saud University, Riyadh 11451, Saudi Arabia

## Abstract

The recent outbreak of the deadly coronavirus disease 19 (COVID-19) pandemic poses serious health concerns around the world. The lack of approved drugs or vaccines continues to be a challenge and further necessitates the discovery of new therapeutic molecules. Computer-aided drug design has helped to expedite the drug discovery and development process by minimizing the cost and time. In this review article, we highlight two important categories of computer-aided drug design (CADD), viz., the ligand-based as well as structured-based drug discovery. Various molecular modeling techniques involved in structure-based drug design are molecular docking and molecular dynamic simulation, whereas ligand-based drug design includes pharmacophore modeling, quantitative structure-activity relationship (QSARs), and artificial intelligence (AI). We have briefly discussed the significance of computer-aided drug design in the context of COVID-19 and how the researchers continue to rely on these computational techniques in the rapid identification of promising drug candidate molecules against various drug targets implicated in the pathogenesis of severe acute respiratory syndrome coronavirus 2 (SARS-CoV-2). The structural elucidation of pharmacological drug targets and the discovery of preclinical drug candidate molecules have accelerated both structure-based as well as ligand-based drug design. This review article will help the clinicians and researchers to exploit the immense potential of computer-aided drug design in designing and identification of drug molecules and thereby helping in the management of fatal disease.

## 1. Introduction

Drug discovery is a lengthy process that takes around 10-15 years [1] and costs up to 2.558 billion USD for a drug to reach the market [2]. It is a multistep process that begins with the identification of suitable drug target, validation of drug target, hit to lead discovery, optimization of lead molecules, and preclinical and clinical studies [3]. Despite the high investments and time incurred for the discovery of new drugs, the success rate through clinical trials is only 13% with a relatively high drug attrition rate [4]. In the majority of the cases (40-60%), the drug failure at a later stage has been reported due to lack of optimum pharmacokinetic properties on absorption, distribution, metabolism, excretion, and toxicity (ADME/Tox) [5]. The use of computer-aided drug discovery (CADD) techniques in preliminary studies by leading pharmaceutical companies and research groups has helped to expedite the drug discovery and development process minimizing the costs and failures in the final stage [6]. The application of rational drug design as an integral part of CADD provides useful insights into the understanding of the binding affinity and molecular interaction between target protein and ligand. Additionally, lead identification in pharmaceutical research has been facilitated by the availability of supercomputing facility, parallel processing, and advanced programs, algorithms, and tools [7]. Furthermore, recent advancements in artificial intelligence (AI) and machine learning methods have greatly aided in analyzing, learning, and explaining the pharmaceutical-related big data in the drug discovery process [8]. Different methods employed in the identification of new inhibitors from chemical databases include pharmacophore modeling, quantitative structure-activity relationship (QSAR), molecular docking, quantum mechanics, and statistical learning methods. CADD can be broadly divided into structure-based and ligand-based drug design approaches, both have been widely used in the drug discovery process in the identification of suitable lead molecules. While the structure-based drug design relies on the three-dimensional structure of the target receptor and its active sites to understand the molecular interaction between the receptor and ligand, the ligand based-drug design depends on the knowledge of ligands interacting with the given target receptor [9]. Computer-aided drug design has a large number of success stories and continues to play a vital role in the drug discovery process [10]. In this regard, the approach has been utilized in proposing drug candidates against coronavirus disease 2019 (COVID-19). COVID-19 is caused by a novel coronavirus known as severe acute respiratory syndrome coronavirus 2 (SARS-CoV-2) which taxonomically belongs to the Betacoronavirus genre and possesses high nucleotide sequence similarity with severe acute respiratory syndrome coronavirus (SARS-CoV) and Middle East respiratory syndrome coronavirus (MERS-CoV). The epidemiology, genome composition, pathogenesis, animal models, diagnostics, and vaccine development with references to various computational biology approaches for MERS-CoV infections have been comprehensively reviewed by Skariyachan et al. (2019) [11]. SARS-CoV-2 is a positive-sense single-stranded enveloped RNA virus approximately 30,000 bp in length which utilizes host cellular machinery to execute various pathogenic processes such as viral entry, genomic replication, and protein synthesis [12].

Like SARS and MERS, the genome of SARS-CoV-2 encodes sixteen nonstructural proteins (nsps) such as main protease (M^pro^), papain-like protease, RNA-dependent RNA polymerase (RdRp), helicase etc., four structural proteins (envelope, membrane, spike, and nucleocapsid), and other accessory proteins. While the spike glycoprotein is essential for the interaction of the virus with the host cell receptor, the nsps play a major role during the virus life cycle by engaging in the production of subgenomic RNAs [13, 14]. The nonstructural and structural proteins, therefore, offer promising targets for the design and development of antiviral agents against COVID-19 [13]. The lack of effective vaccines or drugs for the treatment of COVID-19 and the high mortality rate necessitates the rapid discovery of novel drugs [15], and computer-aided drug design is believed to be an important tool to achieve the identification of novel therapeutics. There is a possibility of the development of effective lead molecules against COVID-19 by utilizing natural lead molecules obtained through virtual screening and pharmacokinetic prediction [16]. To speed up the discovery of a potential treatment for SARS-CoV-2 infection in humans, repurposing of broad-spectrum antiviral drugs is a promising strategy due to the availability of the pharmacokinetic and pharmacodynamic data of these drugs [17]. The availability of complete genome sequence of severe acute respiratory syndrome coronavirus 2 (SARS-CoV-2) and the elucidation of the viral protein structures through X-ray crystallography, nuclear magnetic resonance (NMR), electron microscopy, and homology modelling approach have allowed the identification of inhibitor drugs against the essential therapeutic drug targets of COVID-19. This review article provides useful insights into some of the common in silico methods used in CADD and how these methods have been currently used and can be of help in the drug discovery process of COVID-19.

## 2. Structure-Based Drug Design

**Figure 1:**
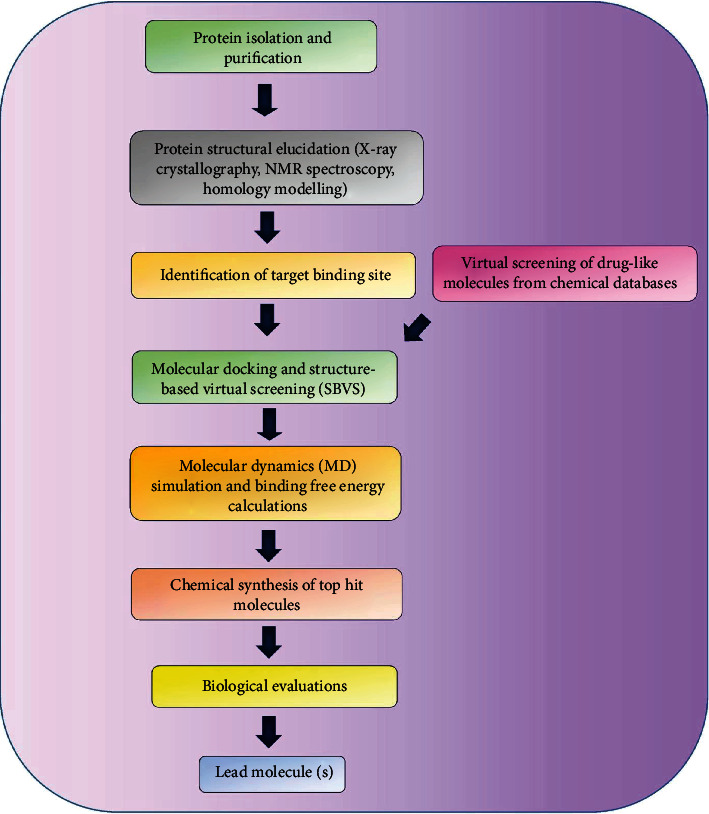
Basic steps involved in the structure-based drug design approach.

The availability of the three-dimensional structure of the therapeutic target proteins and exploration of the binding site cavity forms the basis of structure-based drug design (SBDD) [18]. This approach is specific and effectively fast in the identification of lead molecules and their optimization which has helped to understand disease at a molecular level [19]. Some of the common methods employed in SBDD include structure-based virtual screening (SBVS), molecular docking, and molecular dynamics (MD) simulations. These methods find numerous applications such as assessment of binding energetics, protein-ligand interactions, and conformational changes in the receptor upon binding with a ligand [20]. Being used by many pharmaceutical industries and medicinal chemists, SBDD as a computational technique has greatly helped in the discovery of several drugs available in the market. For example, the discovery of amprenavir as a potential inhibitor of the human immunodeficiency virus (HIV) protease using protein modeling and MD simulations [21, 22], thymidylate synthase inhibitor, raltitrexed against HIV using SBDD approach [23], identification of topoisomerase II and IV inhibitor, norfloxacin which is an antibiotic commonly used against urinary tract infection using SBVS [18], the discovery of dorzolamide, a carbonic anhydrase inhibitor used against glaucoma, cystoid macular oedema using fragment-based screening [24], antituberculosis drug, isoniazid which is an enoyl-acyl-ACP reductase (InhA) inhibitor discovered through structure-based virtual screening and pharmacophore modeling [25], and flurbiprofen, a nonsteroidal anti-inflammatory drug (NSAID) used against rheumatoid arthritis, osteoarthritis etc. which targets cyclooxygenase-2 (COX-2) discovered through molecular docking approach etc. [26, 27]. The basic steps involved in SBDD consist of the preparation of target structure, identification of the ligand binding site, compound library preparation, molecular docking and scoring functions, molecular dynamic simulation, and binding free energy calculation (Figure 1).

### 2.1. Preparation of the Target Structure

With the rapid advancement in structural elucidation techniques such as X-ray and NMR, the structures deposited and available in protein data bank (PDB) have increased over the last few decades. Owing to the limitations of experimental techniques, many target protein structures have not been solved to date [28]. Computational technique such as comparative homology modeling [29], threading [30], and ab initio modeling [31] has been quite successful in deciphering the structures of the proteins from their sequences. Homology modeling is a widely used computational method for accurately determining the three-dimensional structure of a protein from its amino acid sequence using a suitable template structure [32]. It is a multistep process comprising of the following steps: (a) identification of template, (b) sequence alignments, (c) model building of the target (d) model refinement, and (e) model validation [29]. Protein threading is another method for protein structure prediction which is often used when (1) the target protein shares low sequence similarity with other proteins in the PDB (<25% sequence identity), and (2) the target protein shows structural similarity with some proteins in the PDB. Unlike homology modeling, which only takes into account the sequence similarity between the target and the template, protein threading considers the structural information (secondary structure, solvent accessibility and pairwise interactions) encoded in the template to enhance prediction accuracy [33]. The ab initio modeling is another computational technique which is preferably used if the target protein does not have any template structures in the existing biological databases [31]. It considers a global optimization problem to find the dihedral angle values for a given protein structure which contribute to the structure's stability (possessing the global or near global minimum potential energy) [34].

### 2.2. Identification of the Ligand Binding Site

The information about the ligand-binding site is a prerequisite for carrying out specific docking. The knowledge of the binding sites can be extracted from the site-directed mutagenesis study or X-ray crystallographic structures of proteins cocrystallized with substrates or inhibitors [35]. While the experimental information about the binding site of many proteins is not available, there is plenty of software and webservers such as CASTp [36], DoGSite Scorer [37], NSiteMatch [38], DEPTH [39], MSPocket [40], MetaPocket [41], and Q-SiteFinder [42] which allows us to predict the putative binding sites of the target proteins. The bulky compounds which do not fit well within the binding site pocket are rejected during the lead identification procedure.

### 2.3. Compound Library Preparation

Chemical compounds can be selected from chemical databases such as ZINC (N=230 million purchasable compounds) [43], PubChem (N=111 million pure and characterized chemical compounds) [44], MCULE (N=122 million synthetically accessible compounds) (https://mcule.com/), ChEMBL (>1.6 million distinct compounds) [45], DrugBank (N=14528 drug molecules) [46], and ChemSpider ( N=25 million unique chemical compounds) [47]. Molecular docking is performed with drug-like compounds which are filtered using Lipinski's rule of five and ADMET (absorption, distribution, metabolism, excretion, and toxicity) parameters and other risk parameters such as acute rat toxicity, carcinogenicity, serum glutamic oxaloacetic transaminase elevation, hepatotoxicity, and inhibition of 3A4 oxidation of midazolam [28]. According to Lipinski's rule of five, a compound is considered to be orally bioactive if its physicochemical properties lies within the permissible limits such as molecular weight (MW) ≤ 500, partition coefficient between n − octanol and water) (logP) ≤ 5, number of hydrogen bond donor (HBD) ≤ 5, and number of hydrogen bond acceptor (HBA) ≤ 10 [48]. Some commonly used ADMET properties include human gastrointestinal absorption (HIA), blood-brain barrier (BBB) permeation, P-glycoprotein (P-gp) inhibition, cytochromes P450 (CYP) inhibition, and plasma protein binding [49]. Besides the pharmacokinetic properties, drug, and safety, the synthetic accessibility of these compounds should also be taken into account.

### 2.4. Molecular Docking and Scoring Functions

Molecular docking is a computational technique to study the interaction between a target receptor and ligand at the molecular level and allows ranking of the ligands by assessing their binding affinity towards the receptor using various scoring functions [50]. The favorable binding poses of the ligands with a target active site rely on two factors: (a) wide conformational space taking into consideration different binding poses and (b) explicit prediction of binding affinity of ligands corresponding to each binding pose [51]. A list of frequently used molecular docking programs is enumerated in Table 1. Molecular docking can be classified into two types: flexible-ligand search docking and flexible-protein docking. The ligand flexibility in the case of the flexible-ligand search docking method most commonly uses three algorithms such as systematic method, stochastic method, and simulation method [52], whereas flexible-protein docking usually relies on Monte Carlo (MC) and molecular dynamic (MD) methods [53, 54].

### 2.5. Molecular Dynamic (MD) Simulation

The MD simulation of a protein was first performed in the late 1970s [55]. This powerful physical technique is used to predict the positions of each atom in a molecular system with respect to time which is based on Newton's laws of motions governing interatomic interactions [56]. The forces between interacting atoms are estimated using a suitable force field which is used to determine the overall energy of the system [57]. MD simulations have been widely used for several reasons. The position and motion of every atom of the system are captured at every point in time, which is quite tough using any experimental technique. The simulation conditions are exactly known and can be carefully modulated [58]. MD simulations have been extensively used in the structure-based drug discovery process as this technique helps to unravel many atomistic details such as binding, unbinding, and conformational changes in the receptor at a fine resolution which normally cannot be obtained from experimental studies [59, 60]. Further, using MD simulation it is possible to explore the dynamics of receptor-ligand interactions (association and dissociation) and quantify the thermodynamics, kinetics, and free energy landscape [61]. Some examples of MD simulation programs include GROMACS, AMBER, CHARMM, NAMD, and Desmond (Table 2).

## 3. Ligand-Based Drug Design

Ligand-based drug design is another widely used approach used in computer-aided drug design and is employed when the three-dimensional structure of the target receptor is not available. The information derived from a set of active compounds against a specific target receptor can be used in the identification of physicochemical and structural properties responsible for the given biological activity which is based on the fact that structural similarities correspond to similar biological functions [77]. Some of the common techniques used in the ligand-based virtual screening approach include pharmacophore modeling, quantitative structure-activity relationships (QSARs), and artificial intelligence (AI).

### 3.1. Pharmacophore Modeling

A pharmacophore model elucidates the spatial arrangement of chemical features in ligands that are required for interaction with the target receptor [78]. Some of the chemical features used in pharmacophore modeling include hydrogen bond donors, hydrogen bond acceptors, aromatic ring systems, hydrophobic areas, positively charged ionizable groups, and negatively charged ionizable groups [79]. Ligands having different scaffolds but the similar spatial arrangement of key interacting functional moieties can be identified using pharmacophore-based virtual screening. The bioactive conformation of the molecules within the target binding site can be incorporated into the pharmacophore model. The pharmacophore model is also often used in QSAR studies in the molecular alignment stage [80]. Some frequently used programs which allow automatic construction of the pharmacophore model include Catalyst, PHASE, LigandScout, GALAHAD, and PharmMapper (Table 3). A good pharmacophore model also incorporates spatial constraints in regions occupied by inactive molecules and often optimized further to make the model less restrictive. All the pharmacophoric features which are not consistently detected in active molecules are either made optional or removed from the final model [7]. The pharmacophore model generated should have optimum sensitivity and specificity to minimize the chances of false negative and false positive results and must be validated using an independent external test set [81]. If the information about the 3D structure of a receptor and a set of known active compounds are lacking, then a sequence-derived 3D pharmacophore model is quite useful. For example, Pharma^3D^ utilizes knowledge of the 3D crystal structures and homology models to derive the common sequence motif important for receptor-ligand biomolecular interactions in protein families [81, 82].

### 3.2. Quantitative Structure-Activity Relationships (QSARs)

QSAR studies are based on the principle that variations in the bioactivity of the compounds can be correlated with changes in the molecular structures. They are widely used in the drug discovery process in the hit to lead identification or lead optimization. A statistical model is constructed using these correlation studies, and the final model can be used to predict the biological activity of new molecules [80]. The key requirements for the generation of a reliable QSAR model are (a) a sufficient number of data sets with biological activities obtained from common experimental protocols, (b) the training and test set compounds must be appropriately selected, (c) no autocorrelation among the physiochemical properties of the ligands that may cause overfitting of the data, and (d) the applicability and predictivity of the final model must be checked using internal and external validation methods [96]. Based on how the descriptors are derived, QSAR can be classified into six different types: (a) 1D-QSAR which studies the correlation between global molecular properties such as logP and pKa with biological activities, (b) 2D-QSAR wherein biological activities are correlated with the structural patterns such as 2D-pharmacophores and connectivity indices, (c) 3D-QSAR which studies how the biological activities correlated with noncovalent interaction fields surrounding the ligands, (d) 4D-QSAR which is an extension of 3D-QSAR with the addition of an ensemble of ligand configurations, (e) 5D-QSAR which incorporates various induced-fit models in 4D-QSAR, and (f) 6D-QSAR further extends 5D-QSAR by including different solvation models [97]. Some examples of 3D QSAR programs include the HypoGen module of Catalyst [98], PHASE [89], comparative molecular field analysis (CoMFA) [99], and comparative similarity indices analysis (CoMSIA) [100]. A list of tools for the calculation of molecular descriptors is enumerated in Table 4. QSAR technique can be classified into two types: linear and nonlinear based on chemometric methods. The linear method includes linear regression (LR), multiple linear regression (MLR), partial least squares (PLS), principal component analysis (PCA), and principal component regression (PCR). The examples of nonlinear QSAR methods are *k*-nearest neighbours (kNN), artificial neural networks (ANN), and Bayesian neural nets [97].

### 3.3. Artificial Intelligence and Drug Discovery

Artificial intelligence (AI) is a type of machine intelligence that relies on the ability of computers to learn from existing data. AI has been used in various computational modeling methods to predict the biological activities and toxicities of drug molecules [97]. Further, AI has wide applications in drug discovery such as prediction of protein folding, protein-protein interaction, virtual screening, QSAR, evaluation of ADMET properties, and de novo drug design [103]. There are two powerful methods widely used in rational drug design which include machine learning (ML) and deep learning (DL) [104]. ML algorithms that have been extensively used in drug discovery include support vector machine (SVM) [105], Random Forest (RF) [106], and Naive Bayesian (NB) [107]. Few examples of the deep learning methods are convolutional neural network (CNN), deep neural network (DNN), recurrent neural network (RNN), autoencoder, and restricted Boltzmann machine (RBN) [4]. The conventional QSAR methods can efficiently predict simple physicochemical properties such as logP and solubility. However, the QSAR prediction of complex biological properties such as drug efficacy and side effects is often not optimal as the methods use small training sets [108] and has coverage of limited chemical space [109]. The big data generated using high-throughput screening (HTS) techniques are huge challenges to traditional QSAR methods and machine learning techniques [40]. AI methods have been developed to deal with this big data of high volume and multidimensional nature to efficiently predict drug efficacy and side effects in animals or humans. The most promising approach in the present big data world is deep learning which was first used in the drug discovery process in 2012 QSAR machine learning challenge backed by Merck [110]. The results showed that deep learning models were true which can accurately predict the ADMET properties compared to traditional machine learning methods. Although, AI is an impressing method in identification of preclinical candidates in more cost and time-efficient manner, and the accurate prediction of binding affinity between a drug molecule and a receptor using AI remains challenging for quite a several reasons. Firstly, AI is a data mining method whose performance heavily relies on the amount and quality of the available data [4, 111]. Variability in the source of data especially those derived from different biological assays and lack of high-quality data from public databases presents difficulty in efficient AI learning [112, 113].

## 4. Case Study of COVID-19

**Figure 2:**
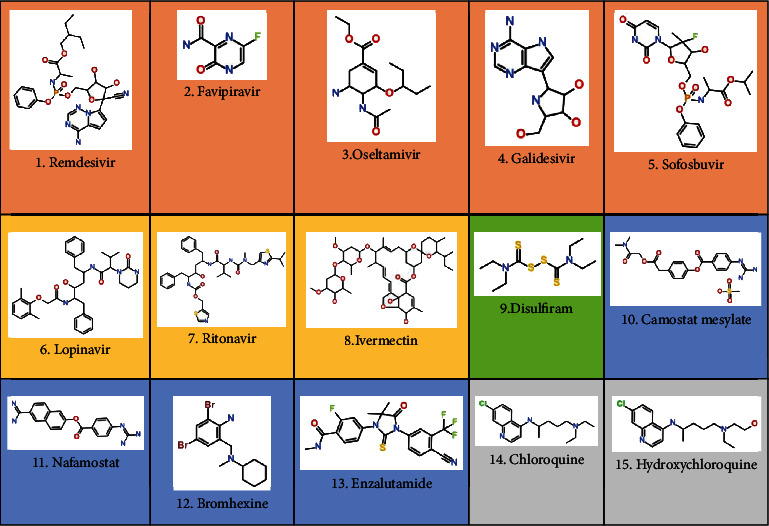
Molecules currently investigated in clinical trials where molecules 1-5 in the orange box are RNA polymerase inhibitors, molecules 6-8 in the yellow box are 3C-like protease inhibitors, molecule 9 in the green box is a papain-like protease inhibitor, molecules 10-13 in the blue box are TMPRSS2 inhibitors, and molecules 14-15 in the grey box are inhibitors of endosomal acidification.

Both ligand-based and structure-based drug design approaches have been widely used in the drug discovery process against coronavirus disease-19 (COVID-19), an infectious viral disease caused by SARS-CoV-2. To date, only a few drug-candidate molecules have undergone clinical trials, and these molecules are mostly repurposed approved drugs (Figure 2).

**Figure 3:**
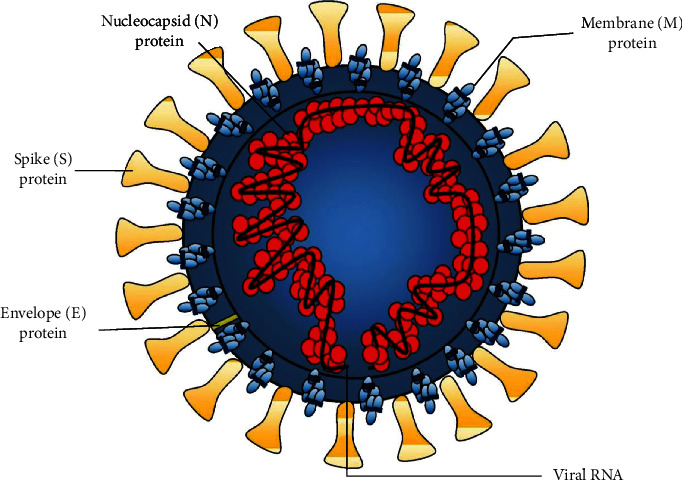
The structural proteins of SARS-CoV-2.

**Figure 4:**
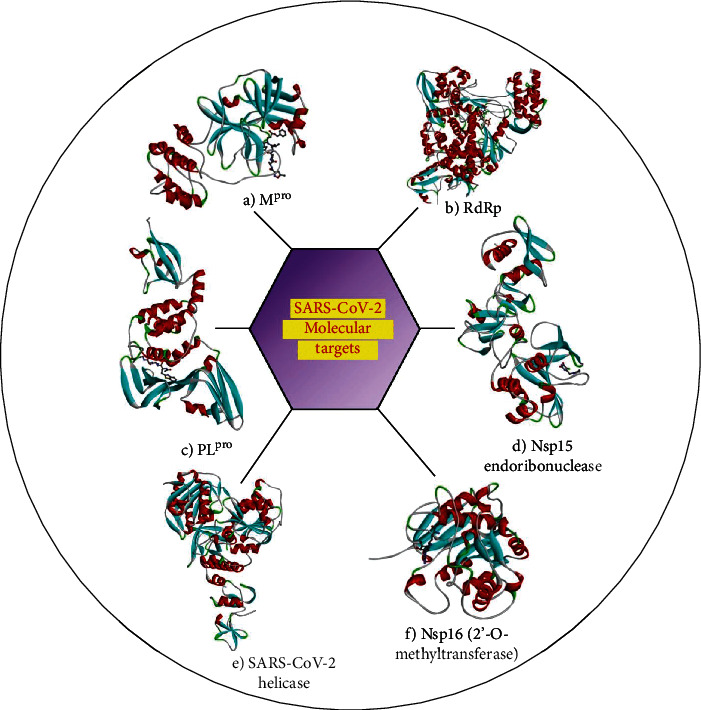
Macromolecular target structures of SARS-CoV-2. (a) X-ray crystal structure of the SARS-CoV-2 main protease in complex with an inhibitor N3 (PDB ID: 7BQY). (b) Crystal structure of Nsp12 (RdRp) bound to triphosphate form of remdesivir (PDB ID: 7BV2). (c) Crystal structure of the SARS-CoV-2 papain-like protease in complex with peptide inhibitor VIR250 (PDB ID: 6WUU). (d) Crystal structure of Nsp15 endoribonuclease from SARS-CoV-2 in complex with potential repurposing drug tipiracil (PDB ID: 6WXC). (e) Crystal structure of the SARS-CoV-2 helicase (PDB ID: 6ZSL). (f) Crystal structure of Nsp16 (2′-O-methyltransferase) from SARS-CoV-2 in complex with sinefungin. The secondary structure elements—helices, sheets, and loops—are colored in red, cyan, and grey, respectively, and the bound inhibitors are rendered as a ball-and-stick model.

The lack of approved drugs and vaccines for COVID-19 and the high mortality rate of the pandemic necessitate identification of effective therapeutics. With the availability of the complete genome sequence of SARS-CoV-2 [114] and structural elucidation of the viral proteins through X-ray crystallography, NMR spectroscopy, electron microscopy and homology modeling, COVID-19 research has been rapidly pursued. Some of the important drug targets of SARS-CoV-2 are the structural protein-spike (S) protein, envelope (E) protein, membrane (M) protein, and the nucleocapsid (N) protein (Figure 3); nonstructural proteins (Nsps) (Figure 4) such as the main protease which is also known as 3C-like protease 3CL^pro^ (nsp5), papain-like protease (PL^pro^, nsp3), RNA-dependent RNA polymerase (RdRp, nsp12), nsp15 endoribonuclease, nsp16 2′-O-methyltransferase, nsp13 helicase, and host-based pharmacological targets are angiotensin-converting enzyme 2 (ACE2), transmembrane protease serine 2 (TMPRSS2), furin, and cathepsin [115]. The details of the nonstructural proteins are briefly discussed here. The main protease is a cysteine protease with a catalytic dyad (cysteine and histidine) in its active pocket [116]. The action of the catalytic activity of M^pro^ on polyproteins results in the release of the vital proteins required for viral replication by cleaving at least 11 sites around the C-terminal and the central regions of the viral polyproteins with sequence consensus X-(L/F/M)-Q↓(G/A/S)-X [117, 118]. Papain-like protease (PLpro) is the second SARS-CoV-2 proteases potentially targetable with small molecules which cleave three sites, with recognition sequence consensus “LXGG↓XX” [118]. It is an attractive drug target because of its essential role in not only the cleavage and maturation of viral polyproteins and assembly of the replicase-transcriptase complex but also disruption of host immune responses [119]. RNA-dependent RNA polymerase (RdRp) is the cleavage product of the polyproteins 1a and 1ab from ORF1a and ORF1ab and is involved in the replication and transcription of the SARS-CoV-2 genome [120]. The catalytic core of the enzyme resembles the human right hand with differentiated palm, fingers, and thumb domains. Targeting this enzyme to halt the viral replication seems an effective therapeutic approach since the active site of the RdRp is a highly conserved and accessible region [121]. Nsp15 is a uridine-specific endoribonuclease involved in RNA processing and widely distributed in all kingdoms of life. Its catalytic C-terminal domain exhibits sequence similarity and functionality of the EndoU family enzymes [122]. The active 234-kDa hexameric enzyme cleaves both single- and double-stranded RNA at uridine sites generating 2′,3′-cyclic phosphodiester and 5′-hydroxyl termini [123]. The SARS CoV-2 2′-o-methyltransferase (nsp16) is another important enzyme target essential for viral multiplication. The enzyme precisely protects the viral RNA from the cellular innate immunity by participating in the formation of a specific arrangement known as RNA cap, a structure which contributes to viral RNA stability and effective process of translation [124]. SARS-Cov-2 Nsp13 helicase is one of the critical enzyme among the 16 known CoV Nsp proteins which shows the highest sequence conservation across the CoV family, indicating their importance for viral multiplication. The enzyme possesses the NTPase and RNA helicase functions that can hydrolyze all types of NTPs and unwind RNA helix in an ATP-dependent process [125]. The transmembrane protease serine 2 (TMPRSS2) is a major host factor which regulates virus-host cell membrane fusion and cell entry by priming of the virus spike (S) protein via cleavage of the S proteins at the S1/S2 and S2 sites [126]. Furin is a type of proprotein convertases (PCs) found in the *trans*-Golgi complex and gets activated by acidic pH. The enzyme recognizes and hydrolyzes the unique “RRAR” motif in SARS-CoV-2-spike protein [127]. Cathepsin L is a lysosomal cysteine protease belonging to a family of proteases involved in proteolysis of protein antigens produced by pathogen endocytosis. The protease cleaves the S1 subunit of the coronavirus spike glycoprotein which is required for the virus entry into human host cells, virus, and host cell endosome membrane fusion [128]. These structures solved through experimental techniques or computational homology modeling techniques can be used for structure-based virtual screening for identification of specific inhibitors of the target proteins.

The CADD methods have been successfully used in the COVID-19 drug discovery process. Selvaraj et al. (2020) solved the three-dimensional structure of SARS-CoV-2 guanine-N7 methyltransferase (nsp14) using the homology modeling method and further proposed five TCM database compounds—TCM 57025, TCM 3495, TCM 5376, TCM 20111, and TCM 31007 as potential antiviral phytochemicals based on molecular docking and simulation studies [129]. Gao et al. (2021) characterized the physicochemical property, subcellular localization, and homology model of the SARS-CoV-2 nucleocapsid protein and further explored its biological function using mass spectrometry analysis and flow cytometry [130]. Beck et al. (2020) used a pretrained deep learning-based drug-target interaction model called molecule transformer drug and identified a few Food and Drug Administration (FDA) approved antiviral drugs such as atazanavir, remdesivir, efavirenz, ritonavir, and dolutegravir showing inhibitory potential against SARS-CoV-2 3C-like proteinase [131]. Elfiky (2020) used homology modeling, molecular dynamic simulations, and molecular docking approaches to target the SARS-CoV-2 RdRp enzyme and reported the suitability of sofosbuvir, ribavirin, galidesivir, remdesivir, favipiravir, cefuroxime, tenofovir, and hydroxychloroquine as candidate drugs for clinical trials [132]. Elmezayen et al. (2020) used a structure-based virtual screening method to identify lead molecules against main proteases and human TMPRSS2. Four potential inhibitors against M^pro^ enzyme identified were talampicillin, lurasidone, ZINC000000702323, and ZINC000012481889, whereas promising inhibitors identified against TMPRSS2 include rubitecan, loprazolam, ZINC000015988935, and ZINC000103558522 [133]. Das et al. (2020) used a molecular docking approach to identify potential inhibitors against SARS-CoV-2 main protease by screening a set of natural products, antivirals, antifungal, antinematodes, and antiprotozoal. The inhibitors identified from the study include rutin (a natural compound), ritonavir (control drug), emetine (antiprotozoal), hesperidin (a natural compound), lopinavir (control drug), and indinavir (antiviral drug) [134]. Gurung et al. (2020) used a molecular docking approach and identified three antiviral phytochemicals: bonducellpin D, 5,7-dimethoxyflavanone-4′-O-*β*-d-glucopyranoside and caesalmin B as potential inhibitors of SARS-CoV-2 M^pro^, SARS-CoV M^pro^, and Middle East respiratory syndrome-coronavirus (MERS-CoV) M^pro^ [135]. Joshi et al. (2020) identified natural molecules such as *δ*-viniferin, myricitrin, taiwanhomoflavone A, lactucopicrin 15-oxalate, nympholide A, afzelin, biorobin, hesperidin, and phyllaemblicin B as potential inhibitors of SARS-CoV-2 M^Pro^ using molecular docking approach [136]. Wahedi et al. (2021) explored stilbenoid analogues as potential anti-COVID-19 drug candidates using molecular docking and molecular dynamic simulation studies and identified piceatannol and resveratrol as important lead molecules for disrupting SARS-CoV-2 and ACE-2 complex formation [137]. Khan et al. (2020) attempted to target chymotrypsin-like protease (3CL^pro^) with small molecules using molecular docking and molecular dynamic simulation approach, and the study revealed three FDA approved drugs (remdesivir, saquinavir, and darunavir) and two natural compounds (flavone and coumarin derivatives) as promising inhibitors of the target enzyme [138]. Further, the potentiality of many dietary flavonols as antiviral drugs targeting the SARS-CoV-2 enzymes and proteins (3CL^pro^, PL^pro^, S protein and RdRp) has been discussed comprehensively by Mouffouk et al. (2021) [139]. Umesh et al. (2021) screened chemical species from Indian spices using a computational approach (molecular docking and molecular dynamic simulation) and identified carnosol, arjunglucoside-I, and rosmanol as potent inhibitors of the novel coronavirus main protease (SARS-CoV-2 M^pro^) [140]. Abdelli et al. (2021) explored essential oil from antiviral and antimicrobial plant *Ammoides verticillata* (Desf.) Briq. that blocks the function of the SARS-CoV-2 angiotensin-converting enzyme 2 (ACE2) receptor using in silico approach (molecular docking, pharmacophore mapping, and MD simulation) and identified isothymol as a promising functional inhibitor of ACE2 receptor [141]. Al-Khafaji et al. (2020) employed a covalent docking screening procedure coupled with the MD simulation technique to identify molecules that can form a covalent bond with Cys145 within the binding pocket of SARS-CoV-2 main protease and identified FDA approved drugs: saquinavir, ritonavir, and remdesivir as top three molecules [142]. Peele et al. (2020) screened FDA approved antiviral drugs, antimalarial drugs, and plant-derived natural drugs with antiviral activity through molecular docking and identified lopinavir, amodiaquine, and theaflavin digallate as promising inhibitors against SARS-CoV-2 main protease and confirmed their stability in the binding pocket of the target enzyme using molecular dynamics simulation [143]. Wang (2020) identified three potential inhibitors of SARS-CoV-2 main protease: carfilzomib, eravacycline, valrubicin, lopinavir, and elbasvir using virtual docking screening of approved drugs and drug candidates in clinical trials followed by MD simulation and binding free energy calculation [144]. Mittal et al. (2021) used antiprotease molecules for drug repurposing against COVID-19 and identified six potential inhibitors of main protease enzyme-leupeptin, hemisulphate, pepstatin A, nelfinavir, birinapant, lypression, and octreotide using virtual screening and molecular dynamic simulation approach [145]. Using molecular modeling and virtual screening approach, Kandeel and Al-Nazami (2020) identified ribavirin and telbivudine as potential inhibitors of SARS-CoV-2 main protease enzyme from a set of FDA approved drugs [146]. ul Qamar et al. (2020) used the homology structure model of SARS-CoV-2 3CL^pro^ for the screening of antiviral phytochemicals and identified three lead compounds 5,7,3′,4′-tetrahydroxy-2′-(3,3-dimethylallyl) isoflavone, myricitrin, and methyl rosmarinate as potential inhibitors of the target enzyme through molecular docking and molecular dynamic simulation approach [147]. Islam et al. (2020) used molecular docking and MD simulation technique and identified five antiviral phytochemicals, viz., hypericin, cyanidin 3-glucoside, baicalin, glabridin, and *α*-ketoamide-11r which showed a good binding affinity with SARS-CoV-2 main protease enzyme [148]. Beura and Chetti (2021) studied few derivatives of chloroquine using pharmacophore modeling, molecular docking, binding free energy calculation, and ADME property analysis and discovered molecule CQD15 as a promising inhibitor of SARS-CoV-2 main protease which shows better interactions with the target enzyme as compared to chloroquine and hydroxychloroquine [149]. Mahanta et al. (2021) screened FDA approved antimicrobial drugs using a combined approach of molecular docking and molecular dynamic simulation and proposed viomycin as a potential inhibitor of the main protease of SARS-CoV-2 [150]. Enmozhi et al. (2021) explored the potentiality of antiviral phytocompound from *Andrographis paniculata* as an SARS-CoV-2 main protease (M^pro^) inhibitor using molecular docking and ADME prediction [151]. Kumar et al. (2020) screened hydroxyethylamine- (HEA-) based library of chemical compounds using molecular docking where HEA is a pharmacophore derived from indinavir. They identified compound 16 as a promising inhibitor of SARS-CoV-2 3CL^pro^ which shows drug-like properties and stable binding within the binding pocket of the target enzyme throughout MD simulation studies [152]. Arun et al. (2020) used the crystal structure of SARS-CoV-2 in complex with an imidazole carboxamide inhibitor and generated an E-pharmacophore hypothesis for the repurposing of drugs. They identified two drugs binifibrate and bamifylline which bind strongly to the enzyme active site pocket as revealed from molecular docking, binding free energy calculation, and molecular dynamic simulation [153]. Gentile et al. (2020) screened marine natural product (MNP) library using hyphenated pharmacophore model, molecular docking, and molecular dynamic simulation approach and identified a total of 17 compounds (belong to the class phlorotannins isolated from the brown alga, *Sargassum spinuligerum*) as potential SARS-CoV-2 M^pro^ inhibitors [154]. Amin et al. (2020) constructed a Monte Carlo optimization-based QSAR model and used it for the virtual screening of some inhouse chemicals. The thirteen identified compounds showed good drug-likeness from SwissADME in silico study, and molecular docking studies further show their favorable interactions with target SARS-CoV-2 PL^pro^, thereby suggesting their potentiality as a seed for drug design and optimization against SARS-CoV-2 PL^pro^ [155]. Similarly, Ghosh et al. (2020) used the Monte Carlo optimization-based QSAR model for screening a library of nature product hits. Fragment analysis of the active molecules suggests that novel potential SARS-CoV-2 M^pro^ enzyme inhibitors may be synthesized by joining fragments/features together or attaching with other scaffolds [156]. Pharmacophore modeling is a fast and effective approach in the identification of interesting lead molecules for drug discovery against COVID-19. A ligand-based pharmacophore model was generated by Law et al. (2020) using established antiviral drugs, and the model was used to estimate the antiviral activity of twenty vanillin derivatives as M^pro^ inhibitors of SARS-CoV-2. Further, the structure-based pharmacophore model suggests that vanillin derivatives (1-20) exhibited promising results, and these compounds were suggested to be potent COVID-19 antiviral compounds [157]. Using the X-ray crystallographic structure of COVID-19 main protease (M^pro^), Daoud et al. (2020) constructed a pharmacophore model and further conducted a molecular docking study to identify antiviral drugs as potential COVID-19 main protease inhibitors. Five FDA-approved antiviral drugs (lopinavir, remdesivir, ritonavir, saquinavir, and raltegravir) were successfully captured by the pharmacophore model, and docking studies revealed that these compounds exhibit many specific binding interactions comparable to that of the cocrystallized inhibitor (X77) [158]. Skariyachan et al. (2020) explored the binding potentiality of six approved drugs (chloroquine, hydroxychloroquine, favipiravir, lopinavir, remdesivir, and ritonavir) against fifteen potential drug targets of SARsS-CoV-2 (spike glycoprotein, RNA dependent RNA polymerase, nsp7, nsp8, papain-like protease, main protease, nucleocapsid protein, heptad repeat of domain 2, ADP ribose phosphatase, nsp9 RNA binding protein, endoribonuclease, orf7a, nsp10, and nsp1) using molecular docking and molecular dynamic simulation approach and concluded that out of all the six drugs, ritonavir and lopinavir showed better binding with the prioritized drug targets [159]. Furthermore, the antiviral mechanisms of these drugs (remdesivir, lopinavir/ritonavir, and chloroquine/hydroxychloroquine) against SARS-CoV-2 have been thoroughly reviewed by Uzunova et al. (2020) [160]. Singh et al. (2021) performed docking-based virtual screening from a library of 1764 antiviral drugs against SARS-CoV-2 NSP12 (RNA polymerase) and identified five compounds, viz., paritaprevir, glecaprevir, velpatasvir, remdesivir, and ribavirin which exhibited high-binding affinity with the drug target [161]. Gowrishankar et al. (2021) screened a total of 57 phytochemicals from three most commonly used Indian herbs (*Justicia adhatoda*, *Eucalyptus globulus*, and *Vitex negundo*) used in “steam inhalation therapy” against four structural protein targets of SARS-CoV-2 viz. 3CL^pro^, ACE2, spike glycoprotein, and RdRp using molecular docking approach, and the best five lead molecules identified were apigenin-o-7-glucuronide, ellagic acid, eudesmol, viridiflorene, vasicolinone, and anisotine [162]. Ibrahim et al. (2021) explored the potentialities of eighteen repurposed drugs in clinical development against SARS-CoV-2 M^pro^ using combined molecular docking and molecular dynamic (MD) techniques and identified TMC-310911 and ritonavir as promising drugs for the treatment of COVID-19 [163].

## 5. Strengths and Challenges of CADD in COVID-19 Research

With the steady rise in the number of confirmed positive and death cases from SARS-CoV-2 infection, computer-aided drug design (CADD) emerges as a fast and reliable technique in pharmaceutical and medicinal research since it not only saves time but also helps to cut costs of designing therapeutic agents [164]. Further, realizing the severity of COVID-19 and the lack of approved therapeutic agents warrants the need for finding potent drugs in less time, and the CADD method makes this possible by facilitating the discovery of new drugs or repurposing FDA-approved drugs whose safety and adverse effects are already known [165]. Since the inherent mutability of the SARS-CoV-2 genome may hinder disease prevention and treatment, CADD can be used efficiently to predict the effects of mutation on drug binding with the molecular receptors [166]. Therefore, CADD can greatly help in accelerating the drug discovery and development process. However, CADD methods have some limitations such as lead molecules derived from the virtual screening process that still need validation through preclinical and clinical assessments before market approval [167]. The fact that the molecular mechanism studies underlying the disease pathogenesis of COVID-19 are still underway, and the existence of bias and imbalance in the limited data available can have a major impact on the prediction accuracy of CADD methods such as artificial intelligence [168].

## 6. Conclusions

Structure-based and ligand-based drug design form two branches of the computer-aided drug discovery process which plays a significant role in the design and identification of drug molecules in reduced time and cost. The increase in the number of positive cases and deaths from COVID-19 and the lack of approved drugs and vaccines continue to be a matter of global health concern which necessitates the urgent discovery of drugs for the prevention and cure of the disease. The structural elucidation of pharmacological targets of SARS-CoV-2 has helped the researchers in the structure-based virtual identification of inhibitors, and the discovery of few lead molecules against COVID-19 has led to the use of scaffolds that can be optimized through ligand-based drug design. Realizing the possible mutability of this RNA virus and the emergence of drug resistance problems, it is, therefore, necessary to take a step further and consider targeting multiple drug targets that will be more effective and might help in overcoming drug resistance barriers.

## Figures and Tables

**Table 1:** Molecular docking tools for protein-ligand interaction studies.

Tools	Key features	Reference
AutoDock	The methods available for conformational searching in AutoDock are Lamarckian genetic algorithm, simulated annealing search, and a traditional genetic algorithm search. The prediction of binding free energies of small molecules to protein targets is based on a semiempirical free energy force field	[62]

AutoDock Vina	AutoDock Vina calculations rely on a sophisticated gradient optimization method and achieve approximately two orders of magnitude improvement in speed and better accuracy of predicting binding modes compared to AutoDock	[63]

GOLD	GOLD (genetic optimization for ligand docking) is an automated ligand docking program that allows full ligand conformational flexibility with partial flexibility of the protein and explores the binding conformations using a genetic algorithm	[64]

CDOCKER	CDOCKER (CHARMm-based DOCKER) is an automated MD docking program that uses the CHARMm19 family of force fields and offers full flexibility of ligand and CHARMm engine with reduced computation time	[65]

FlexX	FLEXX is a full automated docking tool for flexible ligands which produces reliable results with good accuracy. The FlexX method is dependent on the selection and placement of base fragments of ligand and placement and the assumption that the best base fragments interacting with the active site give a good score	[66]

Surflex	Surflex is a docking program that uses a combination of combined Hammerhead's empirical scoring function and molecular similarity method to produce putative poses of ligand fragments	[67]

GLIDE	Glide (grid-based ligand docking with energetics) performs an exhaustive search of the positional, orientational, and conformational space of a ligand binding to a receptor with reasonable computational speed. The scoring of the binding conformations is based on the ChemScore function.	[68]

DOCK6	DOCK 6 is a docking program that evaluates the conformational sampling of small molecules based on the anchor-and-grow search algorithm	[69]

SwissDock	SwissDock is a web server that allows the docking of small molecules to target proteins that are based on the EADock DSS engine	[70]

**Table 2:** A summary of commonly used molecular dynamic (MD) simulation software.

Software	Key features	Simulation system	Reference
GROMACS	GROMACS (Groningen MAchine for chemical simulation) is an efficient and versatile MD program with source code that is suited for the simulation of biological (macro) molecules in aqueous and membrane environments. The program can be run on single processors or parallel computer systems and is compatible with various force fields such as GROMOS, OPLS, AMBER, and ENCAD force fields.	Proteins, lipids, carbohydrate, nucleic acids	[71]

AMBER	Amber is an extensively used biomolecular simulation program with an assembly of codes that are designed to work together. It is a collection of codes that are designed to work together and principally divided into three major step-system preparation (antechamber, LEaP programs), simulation (sander), and trajectory analysis (ptraj analysis program).	Proteins, nucleic acids, carbohydrates	[72]

CHARMM	CHARMM (chemistry at HARvard molecular mechanics) is a widely used molecular simulation program that is primarily designed to study biological molecules such as proteins, peptides, lipids, nucleic acids, carbohydrates, and small molecule ligands. The calculations are based on different energy functions (quantum mechanical-molecular mechanical force fields, all-atom classical potential energy functions) and models such as explicit solvent, implicit solvent, and membrane models.	Proteins, lipids, carbohydrates, nucleic acids	[73]

NAMD	NAMD is a high-performance biomolecular simulation program that employs the prioritized message-driven execution capabilities of the charm++/converse parallel runtime system compatible with parallel supercomputers and workstation clusters.	Proteins, lipids, carbohydrates, nucleic acids,	[74]

Desmond	Desmond is a powerful molecular dynamic simulation program designed by D. E. Shaw with considerable speed, accuracy, and scalability. It supports explicit solvent simulations with periodic boundary conditions and can be used to model explicit membrane systems under various conditions.	Proteins, lipids	https://www.schrodinger.com/desmond

Tinker	Tinker is a molecular modeling and dynamic package written primarily in a standard Fortran 95 with OpenMP extensions. It supports a wide variety of classical molecular simulations particularly biomolecular calculations and offers various force fields including the modern polarizable atomic multipole-based AMOEBA model.	Proteins, nucleic acids	[75]

LAMMPS	LAMMPS (large-scale atomic/molecular massively parallel simulator) is a classical molecular dynamic code for materials modeling. It has potentials for soft matter (biomolecules, polymers), solid-state materials (metals, semiconductors), and coarse-grained or mesoscopic systems.	Proteins, lipids, carbohydrates, nucleic acids	https://lammps.sandia.gov/

DL_POLY	DL_POLY is a general purpose molecular dynamic simulation package, which allows the study of liquids of large complexity. The code is developed using the replicated data (RD) parallelization strategy.	Membranes, proteins	[76]

**Table 3:** A list of pharmacophore modelling tools.

Tools	Description	Reference
Catalyst	Catalyst program is based on an algorithm that identifies three-dimensional configurations of chemical features common to a set of ligands, wherein each configuration is scored based both on estimated rarity and the level to which it is common to the input set.	[83]

LigandScout	LigandScout is a fully automated tool for generating pharmacophore models which detect and classifies protein-ligand interactions (hydrogen bond interactions, charge transfers, and lipophilic regions) which form the basis of the pharmacophore model used for high throughput virtual screening.	[84]

DISCO	DISCO is an automated pharmacophore method which examines the data to find all pharmacophore hypothesis that fit and serves as a complement to 3D QSAR.	[85]

PharmaGist	PharmaGist is a freely available web server used for generating ligand-based pharmacophore models, wherein the input is a set of drug-like molecules (maximum limit of 32 drug-like molecules) that have a binding affinity to the target protein.	[86]

PharmMapper	PharmMapper server is a freely available web server that is commonly used for the identification of potential target receptors for a given small molecule using the pharmacophore mapping approach.	[87]

Pharmer	Pharmer is a pharmacophore search program that organizes molecular data using the Pharmer KDB-tree and bloom fingerprints which allow rapid screening of millions of molecules in a reasonable time.	[88]

PHASE	PHASE is an advanced pharmacophore-based tool that comprehensively maps the common spatial arrangement of functional groups in a set of bioactive ligands using a novel tree-based partitioning algorithm.	[89]

ZINCPharmer	ZINCPharmer is an online web server for the screening of small molecules from the ZINC database using the Pharmer pharmacophore search program. An initial pharmacophore hypothesis can be derived either from PDB structures or by importing pharmacophore models developed using other third-party tools.	[90]

e-Pharmacophore	The e-pharmacophore method generates energetically optimized, structure-based pharmacophore models which can be used for screening of millions of compounds. The method uses the glide XP scoring function to score protein-ligand interactions and has good database screening enrichments.	https://www.schrodinger.com/e-pharmacophores

GASP	GASP program uses a genetic algorithm (GA) for the superimposition of a set of flexible ligands where the ligand possessing the lowest number of chemical features are chosen as a template, onto which other molecules are fitted.	[91]

Shape4	Shape4 is a structure-based pharmacophore program developed is to enhance increase the efficiency of database searching by considering the topographical constraints of the target binding site and consequently helps to help minimize the false positive rate.	[92]

Snooker	Snooker is a structure-based pharmacophore tool that generates pharmacophore hypotheses from homology models with critical residues for ligand binding identified through the study of Shannon entropies of structurally conserved positions in multiple sequence alignments and does not rely on the prior information of ligand structure or interactions.	[93]

Pocketv.2	Pocket v.2 is an automated program to generate a pharmacophore model from a given protein−ligand complex structure and has been designed using the pocket module of LigBuilder.	[94]

GALAHAD	GALAHAD is a pharmacophore program developed to perform flexible alignment of small molecules that bind to a target protein and share similar interaction patterns and shapes.	[95]

**Table 4:** List of programs available for calculating molecular descriptors for building QSAR models.

Programs	Molecular descriptors	Reference
ADMET Predictor	Predicts over 140 properties such as solubility, logP, pKa, sites of CYP metabolism, and Ames mutagenicity.	https://www.simulations-plus.com/software/admetpredictor/

ChemAxon	It provides a wide range of chemical calculations such as molecular weight, elemental composition, LogP, pKa, LogD, LogS, hydrogen bond donor/acceptor (HBDA) count, and various 2D topological descriptors and 3D geometrical descriptors.	https://chemaxon.com/products/calculators-and-predictors

PaDEL-Descriptor	PaDEL-descriptor is a standalone software for calculating molecular descriptors and fingerprints including 797 descriptors (663 1D, 2D descriptors, and 134 3D descriptors) and 10 types of fingerprints.	[101]

E-DRAGON	E-DRAGON is the electronic remote version of the noted software DRAGON, which is an application for the calculation of molecular descriptors (>1,600 molecular descriptors) used for evaluating molecular structure-activity or structure-property relationships.	http://www.vcclab.org/lab/edragon/

DRAGON 7.0	Dragon 7.0 calculates 5,270 molecular descriptors including the simplest atom types, functional groups and fragment counts, topological and geometrical descriptors, and three-dimensional descriptors which are organized into thirty logical blocks.	https://chm.kode-solutions.net/products_dragon.php

CODESSA PRO	CODESSA PRO (comprehensive descriptors for structural and statistical analysis) is an extensive program for studying quantitative structure-activity/property relationships (QSAR/QSPR) by facilitating the calculation of a wide range of molecular descriptors derived from the 2D/3D geometrical structure and/or quantum-chemical wave function of small molecules.	[102]

Pre-ADMET	PreADMET is a web-based program that can be used for the calculation of drug-like physicochemical descriptors such as lipophilicity (logP), molecular weight, polar surface area, and water solubility.	https://preadmet.bmdrc.kr/

QikProp	QikProp program allows the prediction of various pharmacologically important descriptors of chemical compounds such as octanol/water and water/gas log Ps, log S, log BB, overall CNS activity, Caco-2, and MDCK cell permeabilities, log K_HSA_ for human serum albumin binding, etc.	https://www.schrodinger.com/qikprop

ACD/labs	ACD/labs is a multipurpose tool that can be used for the calculation of a variety of physicochemical descriptors such as aqueous solubility, boiling point/vapour pressure, logD, logP, and pKa and ADME properties.	https://www.acdlabs.com/

Corina Symphony	CORINA symphony is a cheminformatic tool for structure representation and calculation of molecular descriptors of six types: global molecular descriptors, shape descriptors, quantum-mechanical properties, 2D property-weighted autocorrelation, 3D property-weighted autocorrelation, property-weighted radial distribution functions (RDF), and autocorrelation of surface properties.	https://www.mn-am.com/products/corinasymphony

MOPAC	MOPAC (molecular orbital PACkage) is a semiempirical molecular orbital package for studying solid-state and molecular structures and reactions and offers calculations of various descriptors of molecules, radicals, ions, and polymers such as the vibrational spectra, thermodynamic parameters, isotopic substitution effects, and force constants.	http://openmopac.net/

## Data Availability

The data is not required.
